# Predictors of transitions in frailty severity and mortality among people aging with HIV

**DOI:** 10.1371/journal.pone.0185352

**Published:** 2017-10-05

**Authors:** Thomas D. Brothers, Susan Kirkland, Olga Theou, Stefano Zona, Andrea Malagoli, Lindsay M. K. Wallace, Chiara Stentarelli, Cristina Mussini, Julian Falutz, Giovanni Guaraldi, Kenneth Rockwood

**Affiliations:** 1 Department of Medicine, Dalhousie University, Halifax, Nova Scotia, Canada; 2 Department of Community Health & Epidemiology, Dalhousie University, Halifax, Nova Scotia, Canada; 3 Division of Geriatric Medicine, Department of Medicine, Dalhousie University, Halifax, Nova Scotia, Canada; 4 Department of Medical and Surgical Sciences for Adults and Children, Clinic of Infectious Diseases, University of Modena and Reggio Emilia, Modena, Emilia-Romagna, Italy; 5 McGill University Hospital Centre, Montreal, Quebec, Canada; 6 Centre for Health Care of the Elderly, Nova Scotia Health Authority, Halifax, Nova Scotia, Canada; Universita degli Studi di Napoli Federico II, ITALY

## Abstract

**Background:**

People aging with HIV show variable health trajectories. Our objective was to identify longitudinal predictors of frailty severity and mortality among a group aging with HIV.

**Methods:**

Exploratory analyses employing a multistate transition model, with data from the prospective Modena HIV Metabolic Clinic Cohort Study, based in Northern Italy, begun in 2004. Participants were followed over four years from their first available visit. We included all 963 participants (mean age 46.8±7.1; 29% female; 89% undetectable HIV viral load; median current CD4 count 549, IQR 405–720; nadir CD4 count 180, 81–280) with four-year data. Frailty was quantified using a 31-item frailty index. Outcomes were frailty index score or mortality at four-year follow-up. Candidate predictor variables were baseline frailty index score, demographic (age, sex), HIV-disease related (undetectable HIV viral load, current CD4+ T-cell count, nadir CD4 count, duration of HIV infection, and duration of antiretroviral therapy [ARV] exposure), and behavioral factors (smoking, injection drug use (IDU), and hepatitis C virus co-infection).

**Results:**

Four-year mortality was 3.0% (n = 29). In multivariable analyses, independent predictors of frailty index at follow-up were baseline frailty index (RR 1.06, 95% CI 1.05–1.07), female sex (RR 0.93, 95% CI 0.87–0.98), nadir CD4 cell count (RR 0.96, 95% CI 0.93–0.99), duration of HIV infection (RR 1.06, 95% CI 1.01–1.12), duration of ARV exposure (RR 1.08, 95% CI 1.02–1.14), and smoking pack-years (1.03, 1.01–1.05). Independent predictors of mortality were baseline frailty index (OR 1.19, 1.02–1.38), current CD4 count (0.34, 0.20–0.60), and IDU (2.89, 1.30–6.42).

**Conclusions:**

Demographic, HIV-disease related, and social and behavioral factors appear to confer risk for changes in frailty severity and mortality among people aging with HIV.

## Introduction

In the era of effective antiretroviral therapy, the number of people aging with HIV is growing. A product of this success, aging with HIV presents new challenges. Treated HIV infection is associated with an increased risk of many non-communicable health problems, including cardiovascular disease, osteoporosis, kidney disease, chronic obstructive pulmonary disease, and cognitive and mobility impairment [1–3]. These conditions span different physiological systems but are all strongly associated with advanced age, leading to the controversial idea that the aging process itself might be accelerated among people with HIV [4]. Potential contributing factors have been proposed, including chronic inflammation, long-term antiretroviral drug toxicity, and associated social and behavioral risk factors [2,5,6].

Understanding aging, and factors that might contribute to differences in the aging process, warrants looking beyond individual age-related diseases to the health of the whole person. Though health generally worsens and diseases accumulate with age, these processes are stochastic, inevitably leading to people of the same chronological age exhibiting differences in overall vulnerability [7,8] including some people who will improve over given, shorter-term intervals. People who are more vulnerable than others of the same age are said to be frailer [9,10]. In this way, frailty can be considered a model of biological (as opposed to chronological) aging [11,12]. Some factors associated with frailty at the same time-point have been identified among people aging with HIV, but few studies have assessed potential predictors of longitudinal changes in frailty severity [6,13,14].

Here, we applied a multistate transition model of changes in frailty severity over a fixed time interval, based on the frailty index, among a cohort people aging with HIV. Using longitudinal data over a four-year interval we sought to identify potential predictors of transitions in frailty severity, including demographic, HIV and treatment-related, and social and behavioral factors.

## Methods

### Setting & sample

This is an exploratory analysis of longitudinal data from the ongoing Modena HIV Metabolic Clinic (MHMC) cohort study, which includes data from patients at the multidisciplinary HIV metabolic clinic at the University of Modena and Reggio Emilia School of Medicine in Modena, Italy. The MHMC cohort was initiated in 2004 to assess metabolic changes among people with HIV aged 18 years and older [15]. Participants undergo an annual study assessment. As this is a clinical study, participants’ data are included in the clinical record, and vital status is updated regularly. Of 2272 enrolled in the longitudinal study, all MHMC participants with four-year follow-up data from their first visit through July, 2014 were included.

### Frailty index

Frailty severity was measured using a frailty index, following the deficit accumulation approach [16–20]. A person’s frailty index score is calculated as the proportion of deficits present out of all health variables considered. (For example, if 40 health variables were available in a database, and a person had 8 health deficits present, their frailty index score would be 8/40 = 0.20). Health variables (including signs, symptoms, laboratory abnormalities, or self-reported health measures) can be included in a frailty index if they meet some basic criteria: variables should characterize acquired health deficits that are generally age-associated, and as a group, should number at least around 30 and cover a range of physiologic systems [9,16,20,21]. The frailty index is robust to the items included, so that in different settings it often incorporates different health variables, and does not require the use of the same variables in each index that is constructed. If the above criteria are met, frailty indices exhibit consistent and reliable characteristics regardless of the individual health variables included–showing typical stochastic dynamics that arise from how deficits interact [7,8]. Frailty index values theoretically range from 0 to 1, although mortality consistently approaches 100% at values around 0.7 (i.e. 2/3 of possible deficits) [18,21]. The frailty index approach has been applied among people as young as 15 years old [22], and in multiple clinical populations including among people living with HIV [23]. The frailty index approach has been compared favorably to other approaches for measuring frailty [24,25], including among people living with HIV [26].

In the present study, thirty-one health variables were selected from the MHMC electronic medical record and re-coded so that they represented the presence or absence of a health deficit (Table 1).

**Table 1:** Health variables included in the frailty index and descriptions of health deficit criteria.

Variable	Description of health deficit
Chronic kidney disease	Two estimated glomerular filtration rate measurements < 60 mL/min/1·73m^2^
NAFLD	Liver/spleen ratio < 1.1
Osteoporosis	Dual-energy x-ray absorptiometry (DEXA) T- or Z-score < -2.5 or fragility fracture
Menopause or male hypogonadism	If female: FSH>30 IU/L & LH<30 IU/L and/or absence of menstruation >1 year; If male: testosterone<300 ng/dL
High or low body mass index	<18 or >25 kg/m^2^
High waist circumference	If female: >88 cm; If male: >102 cm
High visceral adipose tissue (VAT)	VAT>130 cm^2^ or VAT/TAT ratio >0.5
Sarcopenia or presarcopenia	Fat-free mass index < -1 SD
Unemployment	Self-report
Insulin resistance (HOMA)	Homeostasis Model Assessment–Insulin Resistance (HOMA-IR) [51] > 2.8
High total cholesterol	> 200 mg/dL
High low density lipoprotein	>100 mg/dL
Low high density lipoprotein	< 40 mg/dL
High triglycerides	>150 mg/dL
Abnormal leukocyte counts	< 4000 cells/μL
Anemia	If female, < 10 grams/dL; If male, < 12 grams/dL.
Cirrhosis	FIB-4 score [52] > 3.25
Abnormal potassium	< 3.5 or > 5.3 mEq/L
Abnormal phosphorus	< 2.5 or > 5.1 mg/dL
Abnormal thyroid stimulating hormone	< 0.27 or > 4.2 mIU/L
Proteinuria or albuminuria	> 5 mg/mmol
Elevated aspartate transaminase (AST)	> 31 U/L
Elevated alanine transaminase (ALT)	> 31 U/L
Elevated gamma-glutamyl transphosphatase (GGT)	> 55 U/L
Thrombocytopenia	< 150 billion/L
Elevated total bilirubin	> 1·10 mg/dL
Abnormal parathyroid hormone	> 60 pg/mL
Elevated C-reactive protein	> 0.7 mg/L
Vitamin D deficiency	< 30 ng/mL
Lipoatrophy	Multicenter AIDS Cohort Study (MACS) criteria [53]
Lipohypertrophy	MACS criteria

### Covariates

We evaluated the effects on transitions in frailty severity of baseline frailty index, demographic, HIV-related, and social/behavioral factors using generalized linear models [27]. Demographic factors were age and sex. HIV-related factors were current CD4+ T-cell count (indicating the degree of immune recovery with antiretroviral [ARV] treatment), nadir CD4 cell count (indicating the severity of immune depletion before initiation of antiretroviral treatment; low nadir CD4 count is associated with late presentation for care). viral load, duration of HIV infection, and duration of ARV exposure. Social/behavioral factors were pack-years smoking history, history of injection drug use, and hepatitis C virus (HCV) co-infection. Age was categorized by decade. Current and nadir CD4 cell counts were categorized into four clinically relevant groups (<100, 101–250, 251–500, and >500 cells/mm^3^). Viral load was categorized as detectable or undetectable (≤40 copies/mL). Duration of HIV and ARV were categorized by decade. Smoking history was categorized by decades of pack-years.

### Transition model

Probabilities of transitions in frailty severity with age can be summarized using a simple stochastic transition model based on the frailty index [21,27–30]. Although health generally worsens with age, the relationship between aging and health is dynamic and periodic improvement and stability in health are common [9,21,31]. Useful models of biological ageing allow for changes in health that include improvement, maintenance, deterioration, and death [32]. Covariate estimation allows quantification of other factors that might modify such transitions [27].

While individual trajectories of health transitions can appear chaotic, they typically behave in an orderly fashion, and depend on the starting health state–here the number of deficits (i.e. frailty index score) that the person has at baseline. Specifically, as detailed previously [28], the chance of going from any health state (frailty index score at baseline) to a future health state (frailty index score at follow-up) depends first on survival. This is estimated as a logistic function among those who died. The ambient risk of death is estimated by the chance of dying for the people with the fewest deficits at baseline, and the chance of dying increases with the more things people have wrong with them (i.e. the higher their frailty index score). For survivors, the probability of the follow-up health state (frailty index score at follow-up) follows a Poisson distribution and therefore the contribution of multiple covariates can be estimated with a Poission regression, based on the generalized linear model [27,28].

### Analysis

The primary outcomes were transitions in frailty severity and in mortality at four years. The probability of transitions from any frailty index value (number of health deficits out of 31) at baseline to any other value (or death) were assessed using a multistate transition model, described above [27–29]. Each covariate was first evaluated in univariate models, and covariates significantly associated with the outcomes were added into multivariable models. Significance was set at p<0.05. Analyses were performed using SPSS 21·0 (IBM Corp., Armonk, NY). All relevant data are within the paper and its Supporting Information file (S1 Table).

### Ethics committee approval

The research ethics board of the University of Modena and Reggio Emilia provided approval for the MHMC cohort study and all participants provided written consent.

## Results

Of the 2722 participants in the MHMC cohort, 963 had available frailty index scores at baseline and either died or were followed for four years and were included in this study. Participants were generally middle-aged, and just under a third were female (Table 2). Participants generally had high current CD4 cell counts (median 549, interquartile range [IQR] 405–720) and undetectable HIV viral load. Median nadir CD4 was 180 (IQR 81–280). Fifty three percent of participants had a lower frailty index score after four years than at first study visit, while 18.5% maintained the same frailty index score and 28.1% worsened. Mortality was 3·0% (n = 29 deaths).

**Table 2:** Characteristics of study participants.

Sample size (n)	963
Age, years (mean ± SD)	46·8 ± 7·1
Female (%)	29
Current CD4, cells/mm^3^ (mean ± SD)	591 ± 276
Nadir CD4, cells/mm^3^ (mean ± SD)	193 ± 150
Undetectable viral load (%)	89
Duration of HIV infection, years (mean ± SD)	15.0 ± 5.8
Duration of ART, years (mean ± SD)	13.0 ± 9.1
People who inject drugs (%)	27
Hepatitis C virus co-infection (%)	30
Smoking, pack-years (mean ± SD)	16.0 ±15.9

**Fig 1:**
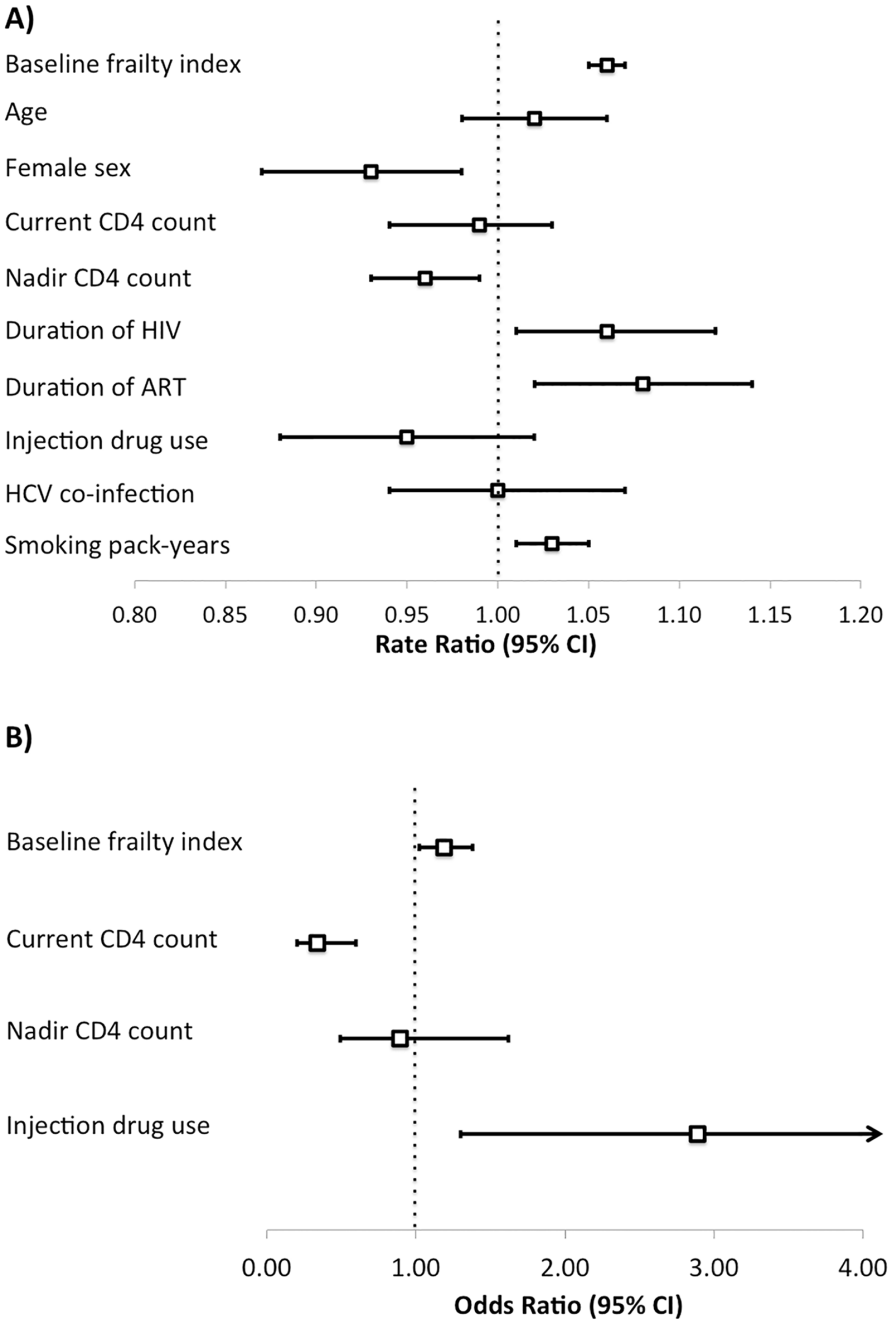
Demographic, HIV-related, and behavioral factors predict changes in frailty and mortality among people aging with HIV. Multivariable analysis of factors associated with (A) frailty index score (per deficit) and with (B) mortality at four-year follow-up in the Modena HIV Metabolic Clinic cohort study.

Baseline frailty index scores strongly influenced transition probabilities and survival. All covariates except for detectable viral load were associated with frailty index scores at follow-up in univariate analyses (Table 3). In multivariable analyses, baseline frailty index (RR 1.06, 95% CI 1.05–1.07), female sex (RR 0.93, 95% CI 0.87–0.98), nadir CD4 cell count (RR 0.96, 95% CI 0.93–0.99), duration of HIV infection (RR 1.06, 95% CI 1.01–1.12), duration of ARV exposure (RR 1.08, 95% CI 1.02–1.14), and smoking pack-years (1.03, 1.01–1.05) were independent predictors of frailty index at follow-up (Fig 1A).

**Table 3:** Univariate predictors of frailty index scores over four years.

Predictors	Rate Ratio	95% CI	p
Baseline frailty index (each deficit)	1.08	1.08–1.09	<0.001
Age (per 10 years)	1.12	1.08–1.15	<0.001
Sex (female)	0·89	0·85–0·94	<0·001
Current CD4 (4 groups)	0·90	0·87–0·93	<0·001
<100 cells/mm^3^	0.91	0.55–1.51	0.7
101–250 cells/mm^3^	1.27	1.16–1.40	<0.001
251–500 cells/mm^3^	1.11	1.06–1.17	<0.001
>500 cells/mm^3^	1.0	-	-
Nadir CD4 (4 groups)	0.88	0.86–0.91	<0.001
<100 cells/mm^3^	1.14	1.00–1.32	0.05
101–250 cells/mm^3^	1.02	0.89–1.17	0.8
251–500 cells/mm^3^	0.86	0.75–0.99	0.03
>500 cells/mm^3^	1	-	-
Viral load (undetectable)	1.02	0.95–1.09	0.7
Duration of HIV infection (per 10 years)	1.16	1.13–1.20	<0·001
Duration of ARV exposure (per 10 years)	1·20	1·15–1·25	<0·001
Injection drug use	1·12	1·07–1·17	<0·001
Hepatitis C virus co-infection	1·11	1·06–1·17	<0·001
Smoking (per 10 pack-years)	1·05	1·04–1·07	<0·001

CD4: CD4+ T-cell count. ARV: antiretroviral. CI: confidence interval.

For predictors of frailty index scores at follow-up, Poisson distributions were used to generate Rate Ratios.

Baseline frailty index, current and nadir CD4 cell count, injection drug use, and HCV co-infection were associated with mortality in univariate analyses (Table 4). In multivariable analyses, independent predictors were baseline frailty index (OR 1.19, 95% CI 1.02–1.38), current CD4 cell count (OR 0.34, 95% CI 0.20–0.60), and injection drug use (2.89, 95% CI 1.30–6.42) (Fig 1B).

**Table 4:** Univariate predictors of mortality over four years.

Predictors	Odds Ratio	95% CI	p
Baseline frailty index (each deficit)	1.33	1.17–1.53	<0.001
Age (per 10 years)	1.43	0.89–2.31	0.1
Sex (female)	0.63	0.25–1.55	0.3
Current CD4 (4 groups)	0.27	0.17–0.44	<0.001
<100 cells/mm^3^	90.17	10.84–750.20	<0.001
101–250 cells/mm^3^	11.27	3.50–36.30	<0.001
251–500 cells/mm^3^	4.03	1.53–10.60	0.005
>500 cells/mm^3^	1.00	-	-
Nadir CD4 (4 groups)*	0.51	0.31–0.85	0.01
Viral load (undetectable)	0.56	0.21–1.49	0.2
Duration of HIV infection (per 10 years)	1.30	0.74–2.30	0.4
Duration of ARV exposure (per 10 years)	0·86	0·42–1·77	0·7
Injection drug use	4·05	1·91–8·61	<0·001
Hepatitis C virus co-infection	2·78	1·27–6·08	0·01
Smoking (per 10 pack-years)	0·67	0·35–1·29	0·2

CD4: CD4+ T-cell count. ARV: antiretroviral. CI: confidence interval.

For predictors of mortality, logistic distributions were used to generate Odds Ratios.

*No deaths occurred in the subgroup with nadir CD4 cell count >500 cells/mm3, and so Odds Ratios for each subgroup were unreliable.

## Discussion

We assessed four-year transitions in frailty severity and mortality among Italians aging with HIV using a multistate modeling approach based on a frailty index, and identified predictors of frailty severity and of mortality. Many predictors were important, with baseline frailty index, sex, current and nadir CD4 cell count, duration of HIV infection, duration of HIV exposure, injection drug use, HCV co-infection and smoking history all associated with outcomes after four years. Frailty is a model of biological aging, and our findings suggest that biological aging is a multifactorial process among people living with HIV. Notably, many of these factors are not specific to HIV infection. Over half of the sample improved in frailty status over four years, and protective factors included female sex, higher current and nadir CD4 cell counts, and fewer smoking pack years. Some factors contributing to frailty might be meaningfully modifiable for individuals and for populations, including baseline frailty, immune status, and behavioral factors.

Our findings should be interpreted with caution. The MHMC cohort includes men and women living in northern Italy who are generally middle-aged, and around a quarter of whom inject drugs, and our findings may not be generalizable to other settings or comparable to some previously published research. For example, frailty has previously been assessed among people living with HIV within cohorts of men who have sex with men [33,34] and within cohorts of people who inject drugs [35,36]. Also, our analysis of morality predictors identified fewer and different factors compared to our analysis of frailty predictors, and included wider confidence intervals. This may be due in part to the relatively few deaths over the follow-up period (which is in turn reflective of the greatly increased life expectancy among people living with treated HIV today), and thereby likely reflects a lack of statistical power. Over longer follow-up further predictors of long-term mortality may be identified.

Recent reviews have summarized what is known about frailty and age-related health conditions in people living with HIV [4–6,13,14,37–39]. Most studies use modified versions of the Fried frailty phenotype instrument [13,40,41], which classifies people as “frail”, “pre-frail”, or “robust”/”not frail”. Few previous studies have applied the frailty index approach in an HIV setting [23,26,42]. As it grades degrees of frailty, the frailty index approach is suitable for investigating what can be subtle changes in how health changes as people age, a benefit that we have aimed to exploit here by situating aging with HIV in the context of aging as deficit accumulation. In cross-sectional analyses, frailty has been associated with demographic, biological, and social factors, including older age, immune system dysfunction, duration of ARV, medical comorbidities, lower education, and lower income [13].

Few longitudinal studies have assessed the progression of frailty among people with HIV [6,13,14]. Escota and colleagues assessed frailty status over time in the SUN study using a modified version of Fried frailty phenotype instrument [43]. Over a median follow-up of 12 months, the majority of participants who were “not frail” at baseline remained “not frail” at follow-up, while the majority of participants who were “frail” at baseline transitioned to “not frail”. Participants who remained “prefrail” or “frail” from baseline to follow-up more often had HCV co-infection, lower median hemoglobin, and higher Veterans Aging Cohort Study index scores [43]. Althoff and colleagues evaluated factors associated with transitioning from not frail to frail between study visits among HIV-positive men who have sex with men in the MACS study [33]. Predictors of transitions included less than college education, having a history of AIDS, and having depressive symptoms, diabetes, or kidney disease. Our findings are consistent with this in that we found the progression of frailty to be multifactorial, including having a low nadir CD4 cell count (or history of AIDS) and HCV co-infection.

Other measures of overall age-related health, associated with frailty, have been assessed in longitudinal studies which identify predictors of biological aging. In the ANRS CO3 Aquitaine Cohort, Richert and colleagues found that middle-aged adults with HIV experienced greater-than-expected declines in six-minute walk test time and five times sit-to-stand (5TSTS) test time [44]. Predictors of decline in 5TSTS test time were history of IDU, cerebral complications of HIV disease, and diabetes. Molsberry and colleagues assessed longitudinal trajectories of cognition in men with and without HIV in the MACS study [45]. They identified heterogeneity in cognitive aging, with a history of AIDS, hepatitis C virus infection, depression, and race all associated with premature cognitive aging.

Our finding that duration of HIV disease and duration of ARV exposure were independently associated with worse health transitions conflicts with a recent report finding no association between these factors and multiple age-related diseases [1]. It is also complicated by the notion that earlier start of ARV, often at higher CD4 counts, is associated with better health outcomes. This is an important area for future research, if ongoing ARV exposure could actually accelerate the aging process in some way. This question of the independent contributions of nadir CD4 cell count and duration of ARV is motivating further inquiry by our group.

A growing body of data from general population and non-HIV clinical settings suggests that the rate of aging is influenced by both extrinsic factors, which determine environmental insults, and intrinsic factors, which determine an organism’s ability to repair damage [27,29]. This was confirmed among people with HIV in the present study, where we found that frailty severity, demographic factors, immune status, duration of HIV infection, duration of ARV exposure, and behavioral factors were all predictive of transitions in health status and/or mortality. The fact that health state transitions, measured by changes in frailty index over time, are multifactorial supports previous cross-sectional data [6,13]. Some of these factors are modifiable, suggesting future opportunities for interventions to prevent or delay the progression of frailty. Other factors may help illustrate the pathophysiology of aging and frailty in people living with HIV and aging in general. For example, nadir CD4+ T-cell count is a predictor of health transitions, suggesting that lasting effects on the immune system from CD4 cell depletion may influence the rate of aging. Clinically, early detection of HIV and initiation of ARV may prevent the lasting effects of immune depletion.

As the HIV-positive population continues to age, understanding of the aging process, risk for age-related diseases, geriatric syndromes, and frailty among people with HIV becomes even more important [39]. Higher rates of many different age-related comorbidities have been identified among people aging with HIV compared to general population rates [1–3,5]. The available evidence suggests that the aging process might differ in some ways among people living with HIV, and might be modestly accelerated in general. People aging with HIV represent a highly heterogeneous group in terms of health status and risk factors [39]. Exploration of the present model of biological aging in other datasets is needed, as well as further longitudinal studies of ageing and health among people with HIV. Further research is needed on the intersection between frailty, HIV, and related, acquired age-associated risk states, such as diabetes [46], COPD [47], social isolation [48], and food insecurity [49,50]. We identified factors across multiple domains that were predictive of health transitions, and not all were specific to HIV infection. Trials to test interventions aimed at recognized risk factors are also needed.

## Supporting information

S1 Table Supporting information file. This table contains the data used in this analysis.
(XLSX)
